# Antibiotic-Induced Dysbiosis of the Gut Microbiota Impairs Gene Expression in Gut-Liver Axis of Mice

**DOI:** 10.3390/genes14071423

**Published:** 2023-07-10

**Authors:** Pu Liu, Yv Zhang, Zhongyuan Zhang, Xiaorong Huang, Xiaojie Su, Shilong Yang, Yongfang Xie

**Affiliations:** Chongqing Key Laboratory of Big Data for Bio Intelligence, Chongqing University of Posts and Telecommunications, Chongqing 400065, Chinas200501017@stu.cqupt.edu.cn (Y.Z.);

**Keywords:** genes, antibiotics, gut microbiota, vitamin C

## Abstract

Antibiotics can be a double-edged sword. The application of broad-spectrum antibiotics leads to the suppression of microorganisms in the human body without selective targeting, including numerous non-pathogenic microorganisms within the gut. As a result, dysbiosis of the gut microbiota can occur. The gut microbiota is a vast and intricate ecosystem that has been connected with various illnesses. Significantly, the gut and liver function in a closely coupled anatomical and physiological relationship referred to as the “gut-liver axis”. Consequently, metabolites stemming from the gut microbiota migrate via the portal vein to the liver, thereby influencing gene expression and proper physiological activity within the liver. This study aimed to investigate the dysbiosis of gut microbiota ecology and the disruption of gene expression resulting from oral antibiotics and their subsequent recovery. In the experiment, mice were tube-fed neomycin (0.5 mg/mL) and ampicillin (1 mg/mL) for 21 days (ABX group) to conduct 16s rRNA sequencing. By simultaneously analyzing public datasets PRJDB6615, which utilized the same antibiotics, it was found that nearly 50% of the total microbiota abundance was attributed to the f__Lactobacillaceae family. Additionally, datasets GSE154465 and GSE159761, using the same antibiotics, were used to screen for differentially expressed genes pre-and post-antibiotic treatment. Quantitative real-time PCR was employed to evaluate gene expression levels before and after antibiotic treatment. It was discovered that oral antibiotics significantly disrupted gene expression in the gut and liver, likely due to the dysregulation of the gut microbiota ecology. Fecal microbiota transplantation (FMT) was found to be an effective method for restoring gut microbiota dysbiosis. To further enhance the restoration of gut microbiota and gene expression, an antioxidant, vitamin C, was added to the FMT process to counteract the oxidative effect of antibiotics on microorganisms. The results showed that FMTs with vitamin C were more effective in restoring gut microbiota and gene expression to the level of the fecal transplant donor.

## 1. Introduction

The colonization of the gut microbiota begins in early life [1] and is often referred to as our ‘invisible organ’ [2]. It is widely accepted that a healthy gut microbiota plays a crucial role in maintaining overall health, while prolonged abnormalities in gut microbiota can lead to metabolic disorders, such as obesity [3], type II diabetes [4], liver cancer [5], and cardiometabolic disease [6]. Although taking antibiotics is commonly used to treat diseases, it can also disrupt the gut microbial ecology and lead to dysbiosis [7,8]. However, supplementing with probiotics can help alleviate this dysbiosis caused by antibiotics [9]. Fecal microbial transplantation (FMT) is an innovative approach to correcting these alterations by transferring fecal microbes from healthy individuals to the patient’s gut [10]. Due to the oxidative effects of antibiotics on microbes [11,12], we speculate that administering antioxidants (such as vitamin C [13]) after antibiotic therapy may aid in the recovery of the gut microbiota. We added vitamin C to the FMT of antibiotic-treated mice (Group VF) and then sequenced 16s rRNA in mouse feces to determine the positive effect of vitamin C on gut microbiota recovery. In addition, we analyzed the external dataset, PRJDB6615, which involved mice treated with the same antibiotics as ours, together with our local data, to explore the gut microbiota characteristics of mice treated with neomycin and ampicillin.

Another important topic to consider is the impact of oral antibiotics on host gene expression through perturbations in the gut microbiota. Research has shown that the gut microbiota can have a significant influence on host gene expression and the epigenetic status of offspring by regulating DNA methylation and RNA methylation modifications [14,15]. Similarly, diet can also affect host gene expression via the gut microbiota, as metabolites released by the gut microbiota can act on histones in tissues such as the proximal colon, liver, and adipose tissue, altering gene transcription through epigenetic pathways [16]. Moreover, a study found that specific innate immune bridging genes, such as *myd88*, play a vital role in regulating immune and host gene expression through the gut microbiota [17].

Furthermore, the gut and liver communicate with each other through the portal vein, biliary tract, and body circulation [18], with approximately 70% of the liver’s blood supply coming from the portal vein. As intestinal venous blood is drained into the portal vein, the liver is the first organ to receive the products and metabolites of the gut microbiota. This relationship between the gut microbiota and the liver has been compared to that of a hen and an egg [19]. Therefore, it is essential to understand how oral antibiotics may disrupt this delicate balance and cause potential long-term impacts on host gene expression and overall health. The gut microbiota has been found to have a significant impact on liver tissue diseases. For instance, in patients with alcoholic liver disease, there is a marked reduction in the proportion of several probiotic bacteria such as *Lactobacillus* spp., *Bifidobacterium* spp., and *Enterococcus faecalis*, as well as a significant decrease in fungal diversity with an absolute predominance of *Candida* spp. [20,21]. Moreover, studies have also revealed a strong association between the gut microbiota and non-alcoholic fatty liver disease [22], cirrhosis [23], hepatocellular carcinoma [24,25], and other diseases related to the enterohepatic axis. Given this close relationship between the liver and gut microbiota, our research included the liver.

To investigate the effects of oral antibiotics on gene expression in the small intestine and liver tissues under the influence of different gut microbiota, we downloaded public datasets, GSE154465 and GSE159761, which used the same antibiotics as our study. We screened these datasets for differentially expressed genes before and after antibiotic treatment and used qRT-PCR to demonstrate differential gene expression in the small intestine and liver tissues. This approach allowed us to examine how oral antibiotics can alter the gut microbiota and affect gene expression in both the small intestine and liver tissues.

**Figure 1:**
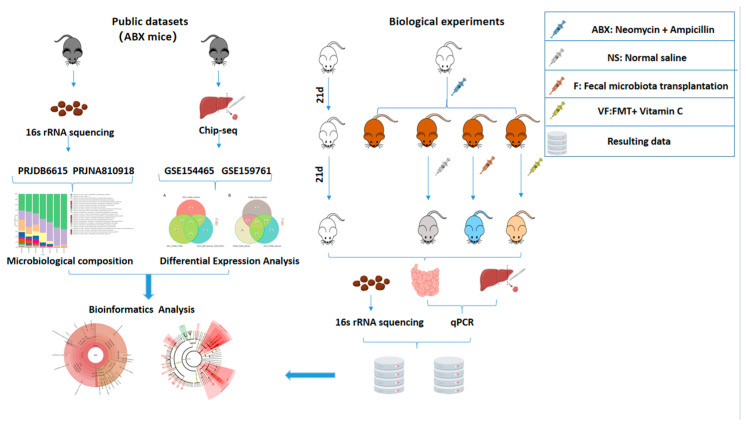
Overall graphical summary.

In summary, our study aimed to investigate the effects of oral neomycin and ampicillin on the composition of the gut microbiota in mice. We used 16s rRNA sequencing to analyze mouse fecal samples and combined our data with a public dataset (PRJDB6615) that also utilized the same antibiotics as ours. Furthermore, we examined the impact of these antibiotics on gene expression in the small intestine and liver tissues using qRT-PCR. Our findings suggest that vitamin C may serve as an adjuvant for restoring the gut microbiota after antibiotic treatment and promoting the recovery of gene expression(Figure 1).

## 2. Materials and Methods

### 2.1. Data Sources

Raw data from local 16s rRNA sequencing were deposited in the NCIB Repository. Accession number: PRJNA879117.

Additional datasets for comparative analysis in this manuscript are available from public databases under accession numbers PRJDB6615, PRJNA810918, GSE154465, and GSE159761.

### 2.2. Animals and Antibiotics

Fifteen C57BL/6J mice (4 weeks old) were purchased from Spelford (Beijing) Biotechnology Co., Ltd. (Beijing, China) and housed at a controlled temperature (23 ± 1 °C) and a 12 h light/dark cycle (lit between 07:00 and 19:00) with sterilized food and water (commercial food pellets were procured from Beijing Keao Xieli Feed Co., Ltd. (Beijing, China)). All experimental procedures were approved by the Research Ethics Committee of Chongqing University of Posts and Telecommunications. Approval reference numbers: 20210324-1.

Mice were tube fed with neomycin (0.5 mg/mL) and ampicillin (1 mg/mL) dissolved in sterile water for 21 days. The mice had free access to the antibiotic water throughout the experiment and it was changed once a day (during this period the mice could only be given antibiotic water). Mice in the control group (group N, *n* = 3) were given drinking water without added antibiotics.

### 2.3. Fecal Microbiota Transplantation

Fresh feces were collected from group N mice. The fecal pellets were mixed with sterile saline (40 mg/mL) and homogenized immediately. The homogenate was centrifuged and the supernatant was collected for transplantation from mice in the ABX group (*n* = 12) that had undergone 21 consecutive days of antibiotic feeding. The ABX mice were randomly divided into three groups, F, VF, and NS, three mice in each group, and the other three were used for qRT-PCR in the ABX group. Mice in group F (*n* = 3) were given 100 μL of fecal supernatant by gavage daily and mice in group VF (*n* = 3) were given 100 μL of vitamin C supplemented fecal supernatant daily (vitamin C dosage of 200 mg/kg). Mice in the NS group (*n* = 3) were given 100 μL of sterile saline daily. We carried out the above for 21 consecutive days. Group N (*n* = 3) mice had free access to food and water.

### 2.4. Conducting 16s rRNA Sequencing

Fecal samples were collected after 21 days of each treatment and all faecal samples were immediately stored at −80 °C in liquid nitrogen. Microbial DNA was extracted from the faecal samples using the HiPure Stool DNA Kit (Magen, Guangzhou, China) according to the manufacturer’s instructions. The highly variable V3-V4 region of the bacterial 16S ribosomal RNA gene was amplified using primers 341F (CCTACGGGNGGCWGCAG), 806R (GGACTACHVGGGTATCTAAT). The PCR cycle conditions are as follows: initial denaturation at 95 °C for 5 min, followed by denaturation at 95 °C for 1 min, annealing at 60 °C for 1 min, extension at 72 °C for 1 min for a total of 30 cycles, and a final extension at 72 °C for 7 min. PCR amplicons were extracted from 2% agarose gels and purified according to the manufacturer’s instructions using the AxyPrep DNA Gel Extraction Kit (Axygen Biosciences, Union City, CA, USA) and quantified using the ABI StepOnePlus Real-Time PCR System (Life Technologies, Foster City, CA, USA). Purified amplicons were double-ended sequenced on the Illumina platform according to standard practice (PE250). The raw Illumina read data were deposited in the NCBI Repository. Accession number: PRJNA879117.

### 2.5. Screening for Differentially Expressed Genes

High-throughput RNA sequencing data were downloaded from the Gene Expression Omnibus (GEO) database (http://www.ncbi.nlm.nih.gov/geo/, accessed on 1 June 2022); we selected datasets, GSE154465 and GSE159761, which used the same antibiotics as we did. GSE154465 contains liver mRNA expression profiles from aged and control-aged mice that were recolonized by two different microbial community types, PAM I and PAM II, after early life exposure to antibiotics, and PAM II-colonized mice had a significantly shorter lifespan compared to PAM I and ABX-free mice. GSE159761 contains liver mRNA expression profiles for day 14 and control in ABX-treated hormonal mice after 14 days. Both datasets use neomycin (0.5 mg/mL) and ampicillin (1 mg/mL) as antibiotic treatments.

Using the latest version of DESeq2 software(1.40.2), the thresholds for significantly different gene expression were set as follows:|log 2 FoldChange (FC)| > 1 and *p*-value < 0.05. Ggplot2 was used to visualize.

GSEA aggregates statistics for each gene in a gene set and can therefore detect when all genes in a predefined set have changed in a small but coordinated way. ID conversion was performed using the R package “Biomart”, “clusterProfiler”. R package “clusterProfiler” was used for gene set enrichment analysis (GSEA). To evaluate several gene features and functional similarities, we used the mclusterSim function in the R package GOSemSim to analyze the overall similarity of the list of gene features relative to their GO annotations. GOSemSim integrates multiple algorithms to calculate functional similarities between gene products based on the information content of GO terms and the similarity of their associated GO annotations.

### 2.6. Quantitative Real-Time-PCR

TRIzol reagent (Invitrogen; Thermo Fisher Scientific, Inc. Shanghai, China) was used, following the instructions. Total RNA was extracted from liver and intestinal tissues. The purity of the RNA was measured using spectrophotometric analysis (Quawell Q3000, USA). The RNA was reverse-transcribed to cDNA using the RevertAid First Strand cDNA Synthesis Kit (Thermo Scientific, USA). We used SYBRPRIME qPCR Kit (Baoguang Biotechnology Co., Ltd., Chongqing, China) and Bio-Rad iQ5 software (Bio-Rad, Shanghai, China) for quantitative real-time PCR (qRT-PCR) analysis. GAPDH was analyzed in each sample to standardize expression. Three biological replicates were performed for each gene, each containing three technical replicates. The primers used in this study are listed in Supplementary Table S5. Relative expression was analyzed using the 2-ΔΔCt method.

### 2.7. Bioinformatics Analysis

FASTP (version 0.18.0) filters raw data from the Illumina platform, using FLASH to merge paired ends. Sequence analysis was performed using UPARSE (version 9.2.64) and the UCHIME algorithm to select the most abundant tag sequence as representative of each OTU. Species classification was annotated using the SILVA database (version 132) and a plain Bayesian model with RDP annotation software (version 2.2), with confidence thresholds set at 0.8~1. Krona (version 2.6), was used to display the abundance statistics for each species classification. Biomarker species from each group were screened using LEfSe software [26] (version 1.0), the randomforest package (version 4.6.12), the pROC package (version 1.10.0), and the labdsv package (version 2.0-1). Diversity indices were calculated in QIIME [27] (version 1.9.1). PCoA (principal coordinates analysis) was based on weighted unifrac distances using the Vegan package (version 2.5.3). KEGG (Kyoto Encyclopedia of Genes and Genomes) metabolic pathway analysis of sample bacteria or archaea was performed using PICRUSt 2. Classification of microbial phenotypes of bacteria was carried out using BugBase [28]. Differentially expressed genes were screened using DESeq2 software (|log 2 FoldChange (FC)| > 1 and *p*-value < 0.05).

### 2.8. Statistical Analysis

*p* < 0.05 was considered statistically significant when comparing any two groups using the Student *t*-test.

## 3. Results

### 3.1. Significant Changes in Gut Microbiota Composition at the Family and Genus Level in Mice after Oral Antibiotic Dosing

**Figure 2:**
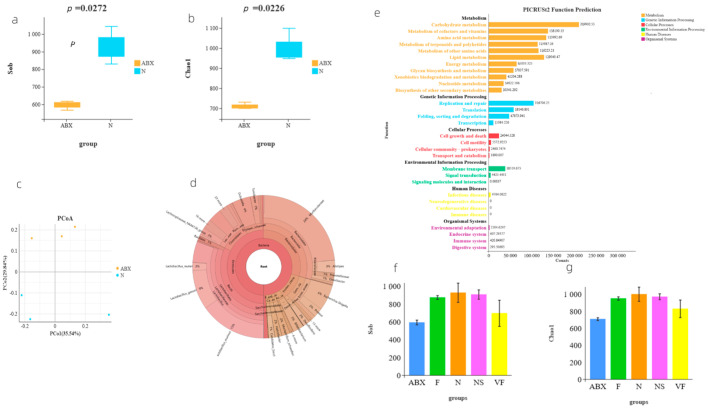
(**a**) Comparison of *α* diversity based on the Sob index; (**b**) comparison of *α* diversity based on the Chao1 index; (**c**) PCoA analysis of the community composition of samples from groups ABX and N; (**d**) Krona pie chart of species for a sample of N groups; (**e**) functional prediction of microorganisms with PICRUSt2; (**f**) comparison of Sob-based *α* diversity between arbitrary groups; and (**g**) comparison of Chao1-based *α* diversity between arbitrary groups.

Our analysis of *α* diversity using the Sob (*p* = 0.0272) and Chao1 (*p* = 0.0226) indices revealed significant differences in gut microbiota abundance between the ABX-treated mice and the N group, indicating the significant inhibitory effect of antibiotics on microbiota abundance (Figure 2a,b). To further demonstrate the differences in taxonomic composition between the two groups, we utilized principal coordinates analysis (PCoA analysis), which clearly showed the complete separation of the samples from the ABX and N groups (Figure 2c).

To gain insight into the species composition of gut microbiota after antibiotic treatment, we downloaded 16s rRNA sequencing data from the public database (PRJDB6615) for the same antibiotic used in our study. At the family level, both our local data (54.59% relative abundance of f__Lactobacillaceae) and the PRJDB6615 dataset (47.17% relative abundance of f__Lactobacillaceae) were dominated by f__Lactobacillaceae, followed by f__Muribaculaceae (average 18.78%) and f__Erysipelotrichaceae (average 7.78%) (Supplementary Tables S1 and S2). Similarly, at the genus level, g__Lactobacillus was the most dominant genus in both our local data (42.19% relative abundance of g__Lactobacillus) and the PRJDB6615 dataset (47.17% relative abundance of g__Lactobacillus). In our local data, this was followed by g__Muribaculaceae (27.58%), g__Bifidobacterium (9.12%), whereas, in the PRJDB6615 dataset, it was followed by g__Allobaculum (7.01%) and g__Muribaculaceae (5.90%) (Supplementary Tables S3 and S4).

In the N group mice, which had a more balanced and diverse distribution of gut microbiota species, f__Lactobacillaceae still accounted for a large proportion (19.32%), with f__Muribaculaceae having the highest relative abundance (36.16%) (Figure 2d).

To summarize, prior to antibiotic treatment, f__Muribaculaceae comprised the largest proportion (36.16%) of gut microbiota in mice, followed by f__Lactobacillaceae (19.32%). However, following antibiotic administration, f__Lactobacillaceae became the most abundant species, accounting for approximately 50% of the total, likely due to the inhibition of other microorganisms by the antibiotics.

**Figure 3:**
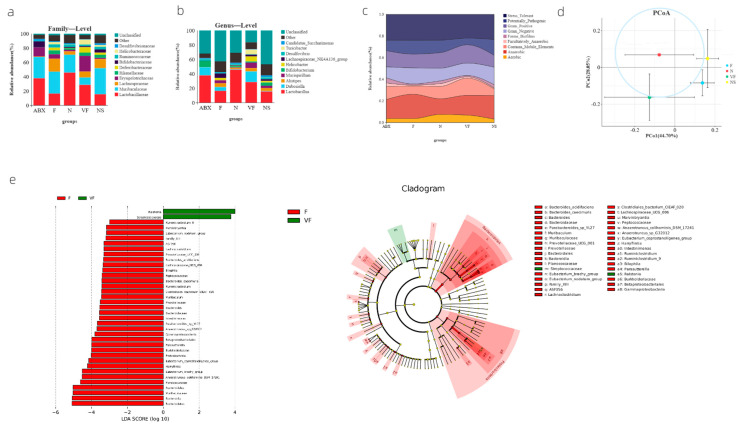
(**a**) Species composition at family level in each group; (**b**) species composition at genus level in each group; (**c**) river diagram of the results of microbial phenotyping analysis results; (**d**) weighted UniFrac based PCoA results; and (**e**) LEfSe results based on indicator species.

We utilized PICRUSt2 to predict the functions of the microbiota genes that remained colonized in the mouse intestine after receiving ABX, and found that their functions were focused on metabolism and genetic information processing, with other functional pathways accounting for a relatively small proportion (Figure 2e). Additionally, BugBase phenotypic analysis indicated that the phenotypes of the microbiota after receiving ABX treatment were not significantly different from those of the N group (Figure 3c).

### 3.2. Weighted UniFrac-Based PCoA Results Show That Vitamin C Contributes to the Recovery of Gut Microbiota to the Donor

**Figure 4:**
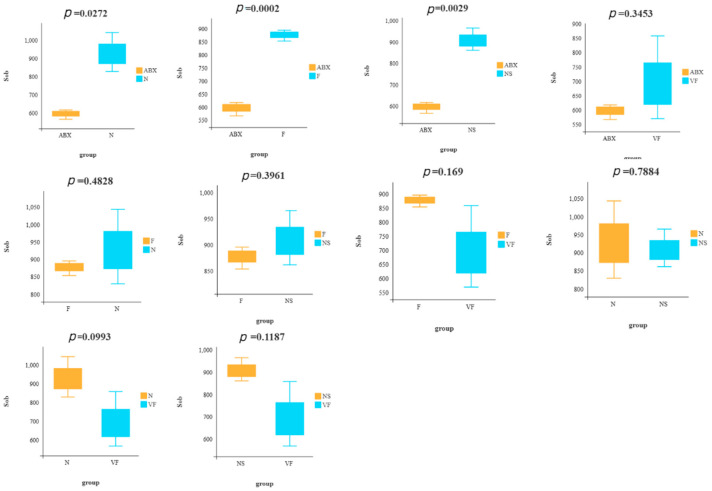
Comparison of Sob-based *α* diversity between any two groups.

Our analysis of the public database of 16s rRNA data (PRJNA810918) revealed that mice receiving 8 consecutive weeks of FMT from mice not treated with antibiotics still did not return to normal mouse levels of *α* diversity (Supplementary Figure S1). To assess the recovery of gut microbiota in each group, we utilized Sob- and Chao1-based *α* diversity and performed a *t*-test for Sob-based *α* diversity in any two groups (Figure 4). Our results showed that, in terms of diversity alone, the NS group exhibited the best recovery, followed by the F and VF groups. However, after 21 consecutive days of treatment, the *α* diversity was still not as good in either the F, NS, or VF groups as in the N group (Figure 2f,g). This suggests that the recovery of the gut microbiota after oral antibiotic administration may take a considerable amount of time.

At both the family and genus level, we observed a significant decrease in the proportion of f__Muribaculaceae (*p* = 0.0381) and a significant increase in the proportion of g__mucispirillum (*p* = 0.0003085) in the VF group compared to the F group (Figure 3a,b). Furthermore, our bugBase phenotypic analysis showed a significant increase in Aerobic (*p* = 0.02981) and a significant decrease in Potentially_Pathogenic (*p* = 0.02144) in the VF group compared to the F group. Comparing the VF group to the NS group, the VF group was significantly more Aerobic (*p* = 0.02259) and significantly less Potentially_Pathogenic (*p* = 0.00537). In contrast, while there was no significant difference in the ABX group compared to the N group, we did observe a significant increase in Stress_Tolerant when comparing the ABX group to the F group (*p* = 0.00115) (Figure 3c). Linear Discriminant Analysis Effect Size (LEfSe) analysis, which was based on indicator species, revealed species with significantly different abundances between the two groups [LDA score (log10) > 2]. The VF group exhibited significantly higher abundances of Ralstonia and Streptococcacease, while the remaining indicator species were all more abundant in the F group (Figure 3e). Additionally, we conducted Weighted UniFrac PCoA to assess both species diversity and abundance, and found that the closest group to the N group was VF, followed by NS and F (Figure 3d).

### 3.3. Screening for Six Differentially Expressed Genes before and after Antibiotic Treatment

**Figure 5:**
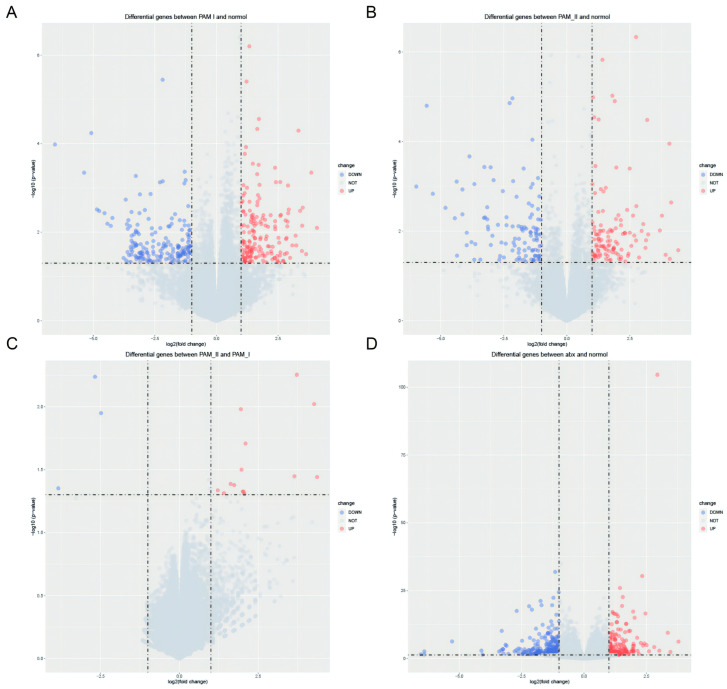
(**A**) Differential analysis of PAM I colonization in GSE154465 compared to ABX-free mice. (**B**) Differential analysis of PAM II colonization in GSE154465 compared to ABX-free mice. (**C**) Differential analysis of PAM II colonization in GSE154465 compared to PAM I colonized mice. (**D**) Differential analysis of ABX-treated tumor-bearing mice in GSE159761 compared to ABX-free mice.

To investigate the effects of antibiotic treatment on gene expression, we obtained datasets GSE154465 [29] and GSE159761 [30] from public databases, which employed neomycin (0.5 mg/mL) and ampicillin (1 mg/mL) as antibiotics, the same as those used in our study. We verified the differential gene expression following antibiotic treatment through the utilization of Quantitative Real-time-PCR. In GSE154465, we found 343 homologous genes that were significantly differentially expressed in PAM I-colonized mice compared to normal ABX-free mice, with 168 exhibiting high expression and 175 displaying low expression (Figure 5A). Similarly, we observed 223 differentially expressed homologous genes in PAM II-colonized mice relative to normal ABX-free mice, 114 of which exhibited high expression and 109 displayed low expression (Figure 5B). Seventeen homologous genes were significantly differentially expressed in PAM II-colonized mice compared to PAM I-colonized mice, of which 14 were highly expressed genes and 3 were lowly expressed genes(Figure 5C). In GSE159761, ABX-treated tumor-bearing mice exhibited significant differential expression of 412 homologous genes compared to normal mice, including 188 highly expressed genes and 224 lowly dexpressed genes (Figure 5D).

**Figure 6:**
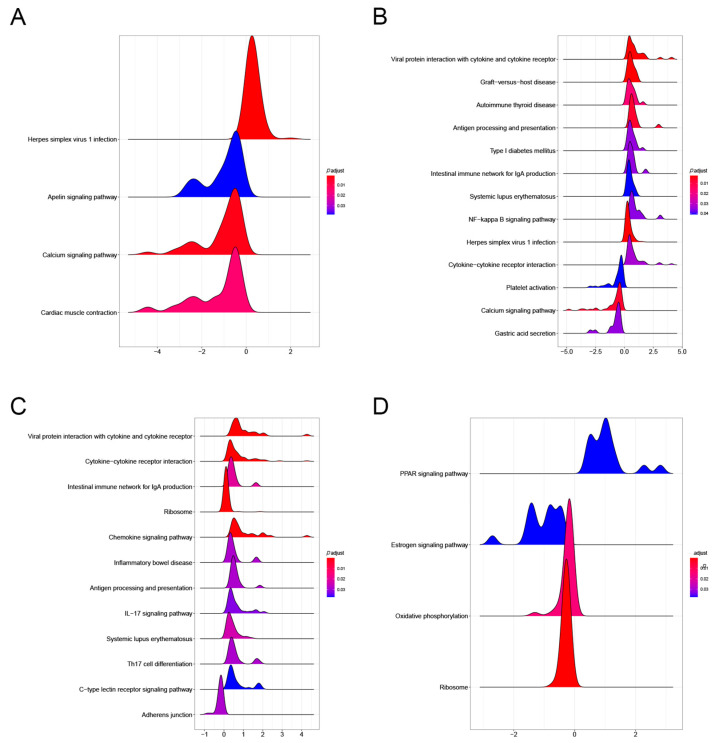
(**A**) KEGG pathway enrichment results of PAM I colonization in GSE154465 compared to ABX-free mice. (**B**) KEGG pathway enrichment results of PAM II colonization compared to ABX-free mice.(**C**) KEGG pathway enrichment results in PAM II colonized compared to PAMI colonized mice. (**D**) KEGG pathway enrichment results in tumor-bearing mice compared to normal mice in GSE159761.

KEGG results showed that PAM I colonization in GSE154465 was mainly enriched in four pathways, namely the Herpes simplex virus 1 infection, Calcium signaling pathway, Cardiac muscle contraction, and Apelin signaling pathway, compared to normal ABX-free mice (Figure 6A). PAM II colonization, compared to normal ABX-free mice, was mainly enriched to Viral protein interaction with cytokine and cytokine receptor, Herpes simplex virus 1 infection, Calcium signaling pathway, and 13 other pathways (Figure 6B). The major enrichment pathways, Herpes simplex virus 1 infection, Viral protein interaction with cytokine and cytokine receptor, and Herpes simplex virus 1 infection, are all immune-related. PAM II colonization was enriched in 12 pathways, including cytokine–cytokine receptor interaction, Viral protein interaction with cytokine and cytokine receptor, and Ribosome, compared to PAM I-colonized mice (Figure 6C). In contrast, the tumor-bearing mice in GSE159761 were mainly enriched in four pathways, including Ribosome, Oxidative phosphorylation, and others, compared to normal mice (Figure 6D).

**Figure 7:**
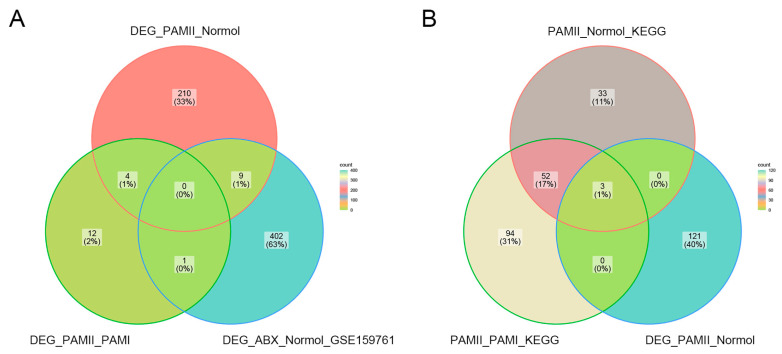
(**A**) Differential genes in GSE154465 for PAM II-colonized compared to ABX-free mice and PAM II-colonized compared to PAM I-colonized mice and in GSE159761 for ABX-treated tumor-bearing mice compared to normal mice. (**B**) KEGG pathway gene crosses in PAM II-colonized compared to PAM I-colonized mice and ABX-free mice and differential gene crosses in PAM II-colonized compared to ABX-free mice.

We focused on the data from PAM II-colonized mice because of the significantly shorter lifespan compared to PAM I and ABX-free mice. There were four intersections of differential genes in PAM II-colonized compared to PAM I-colonized mice or normal ABX-free mice (Figure 7A). A total of nine genes were intersected between the differential genes in ABX-treated hormonal mice versus normal mice in GSE159761 for the PAM II-colonized phase and PAM I colonized mice compared to normal ABX-free mice (Figure 7A). During the comparison between PAM II-fixed compared to PAM I-fixed mice, and PAM II-fixed compared to normal ABX-free mice, 55 core genes were repeated in both, with Cd4, Ccr3, and Ccr9 being the genes that were significantly different in PAM II-fixed compared to normal ABX-free mice (Figure 7B). Deleting genes that could not be mapped to human homologs (via the R package Biomart), and genes with uncertain functionality or that were associated with multiple complex functions, we finally selected six genes, Cd4, Ccr3, Ccr9, Tenm4, Lonrf3, and Rgs16, as the genes to be investigated.

### 3.4. Gut Microbiota Significantly Affects Gene Expression in the Gut-Liver Axis, which FMT with Vitamin C May Help to Restore

To investigate the perturbation of gene expression in mice by ABX treatment and the recovery of each group, we examined the changes in the expression of these six genes in the small intestine and liver of mice in the ABX, VF, F, NS, and N groups by qPCR.

#### 3.4.1. Gut Microbiota Significantly Affect Gene Expression in the Liver

**Figure 8:**
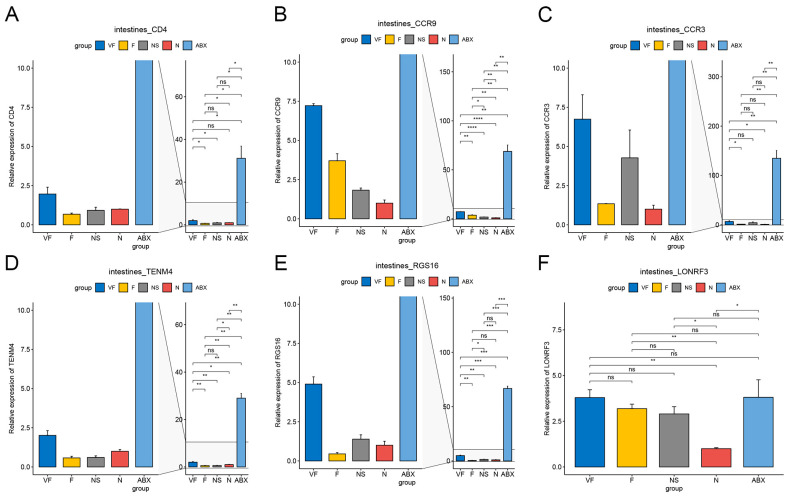
qPCR results of 6 genes in intestine with different treatments. (**A**) Cd4, (**B**) Ccr9, (**C**) Ccr3, (**D**) Tenm4, (**E**) Rgs16, and (**F**) Lonrf3. (*: *p* < 0.05,**: *p* < 0.01,***: *p* < 0.001).

**Figure 9:**
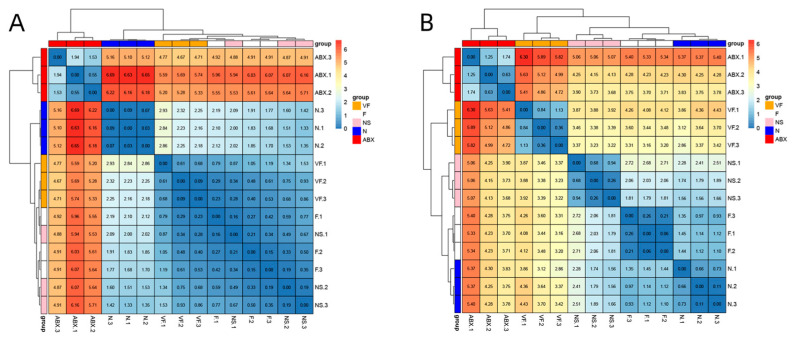
(**A**) Clustered heat map of gene expression in the small intestine. (**B**) Clustered heatmap of gene expression in the liver.

The ABX group showed a significant increase in the expression of all six genes relative to the N group and the effect of oral antibiotics on gene expression in the gut was quite significant. After 21 days of cessation of ABX treatment, the NS group showed a significant decrease in Cd4, Ccr9, Ccr3, Tenm4, and Rgs16 relative to the ABX group, while Lonrf3 showed no significant difference in change. After transplanting the feces of healthy mice for 21 consecutive days, Cd4, Tenm4 significantly decreased and Ccr9, Lonrf3 significantly increased in group F compared to group N. Compared to group NS, Ccr9 significantly increased and Rgs16 significantly decreased in group F. There were no significant differences in other genes. After additional vitamin C was added during fecal transplantation, all six gene expressions were significantly increased in the VF group relative to the N group, and all five genes were significantly increased relative to the F group, with the exception of Lonrf3, which was not significantly increased (Figure 8). The clustering results based on Euclidean distance showed that the ABX group clustered together individually and that the VF group was the closest to the N group apart from the ABX group (Figure 9A).

#### 3.4.2. Gut Microbiota Significantly Affect Gene Expression in the Liver

**Figure 10:**
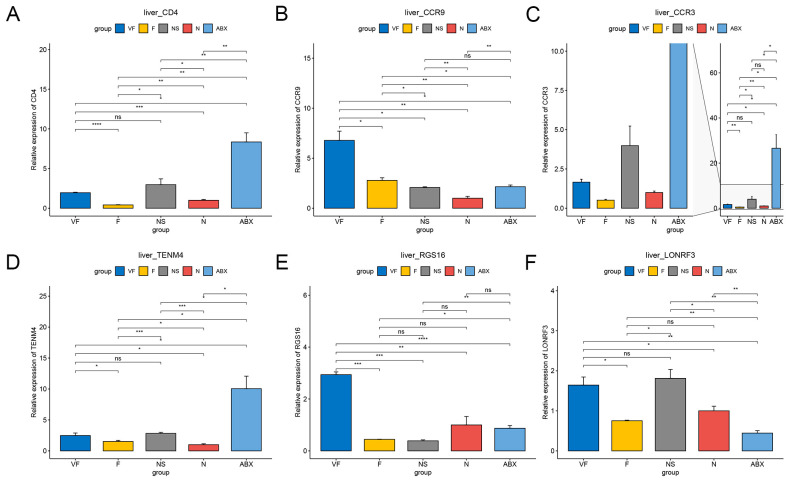
qPCR results for 6 genes in liver with different treatments. (**A**) Cd4, (**B**) Ccr9, (**c**) Ccr3, (**D**) Tenm4, (**E**) Rgs16, and (**F**) Lonrf3. (*: *p* < 0.05,**: *p* < 0.01,***: *p* < 0.001).

ABX treatment significantly increased Cd4, Ccr3, Ccr9, and Tenm4 expression relative to the N group, while Lonrf3 significantly decreased. After 21 days of stopping ABX treatment, Cd4, Ccr3, Tenm4, and Rgs16 decreased significantly and Lonrf3 increased significantly in the NS group relative to the ABX group. Cd4, Ccr9, Tenm4, and Lonrf3 were significantly upregulated in the NS group compared to the N group. Changes in Ccr3 and Rgs16, although not significant, were higher in Ccr3 than in the normal group and lower in Rgs16 than in the normal group. These were generally consistent with the results of the GSE154465 dataset. After transplanting feces from healthy mice for 21 consecutive days, the F group was closer to normal mice than the NS group for five genes except for Ccr9 (which was significantly higher). Compared to group N, group F showed a significant decrease in Cd4, Ccr3, and a significant increase in Ccr9, Tenm4, while no significant differences were found in other genes. After additional vitamin C was added during fecal transplantation, the expression of all six genes was significantly increased in the VF group relative to the F and N groups (Figure 10). The clustering results based on Euclidean distance showed that group F was the closest to group N (Figure 9B).

#### 3.4.3. Gut Microbiota also have a Significant Effect on the Expression of Other Genes in the Gut-Liver Axis

As the two microarrays, GSE154465 and GSE159761, did not cover all genes, we tried to select some representative genes outside their datasets for testing. Seven genes were selected for study, namely TNF-α, Srebp-1, Mfns, LXR, IL-6R, IL-1β, and Chrebp, which are involved in inflammatory cytokines, lipid synthesis, mitochondrial fusion, lipid and cholesterol metabolism, immunity, carbohydrate response, and many others.

The qPCR results showed that these seven genes were significantly decreased in the liver in the ABX group compared to the N group of normal mice, unlike in the intestine where they were significantly increased. After 21 days of stopping ABX treatment, TNF-α, Srebp-1, Mfns, IL-6R, and IL-1β were still decreasing in the NS group compared to the ABX group, while LXR was significantly increasing and Chrebp was not significantly different, and seven genes were still significantly decreased in the NS group compared to the N group. In the intestine, TNF α, Srebp-1, Mfns, and IL-1β were significantly decreased in the NS group compared to the ABX group, while Chrebp, LXR, and IL-6R were not significantly changed. Compared to the N group, Mfns, LXR, IL-1β, and Chrebp increased significantly in the NS group, while the remaining genes did not change significantly.

After transplantation of healthy gut microbiota, Srebp-1, Mfns, LXR, IL-6R, IL-1β, and Chrebp were significantly increased in liver tissue in the F group compared to the NS group, with no difference in TNF-α. Overall it was closer to the N group, but still significantly lower in TNF-α, Srebp-1, Mfns, IL-6R, and IL-1β compared to the N group. In the intestine, the trends in these genes were similar to those in the liver compared to the NS group. However, all six genes were significantly increased compared to the N group except for TNF.

**Figure 11:**
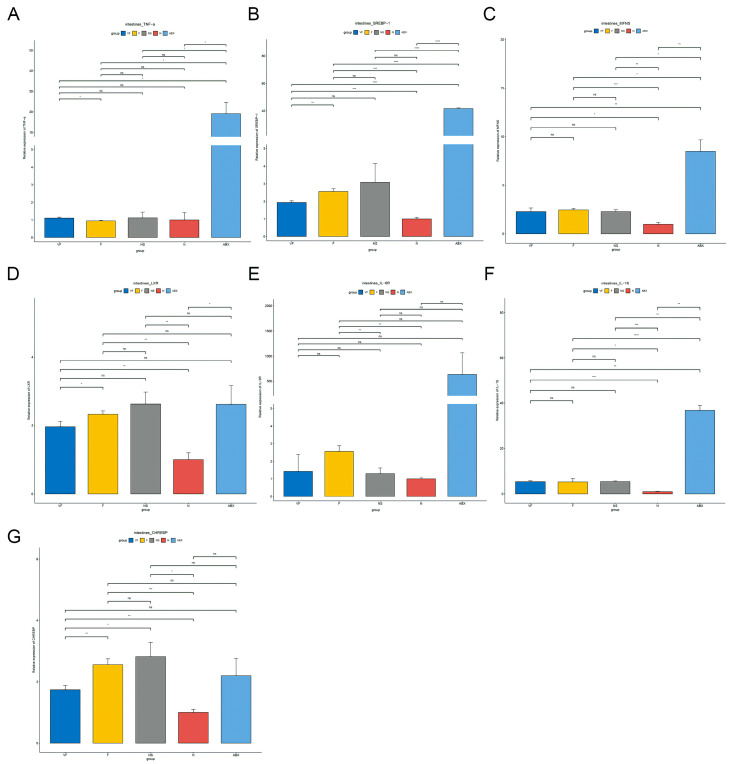
Gene qPCR results from different treated mice in the gut. (**A**) TNF-α, (**B**) Srebp-1, (**C**) Mfns, (**D**) LXR, (**E**) IL-6R, (**f**) IL-1β, and (**G**) Chrebp. (*: *p* < 0.05,**: *p* < 0.01,***: *p* < 0.001).

**Figure 12:**
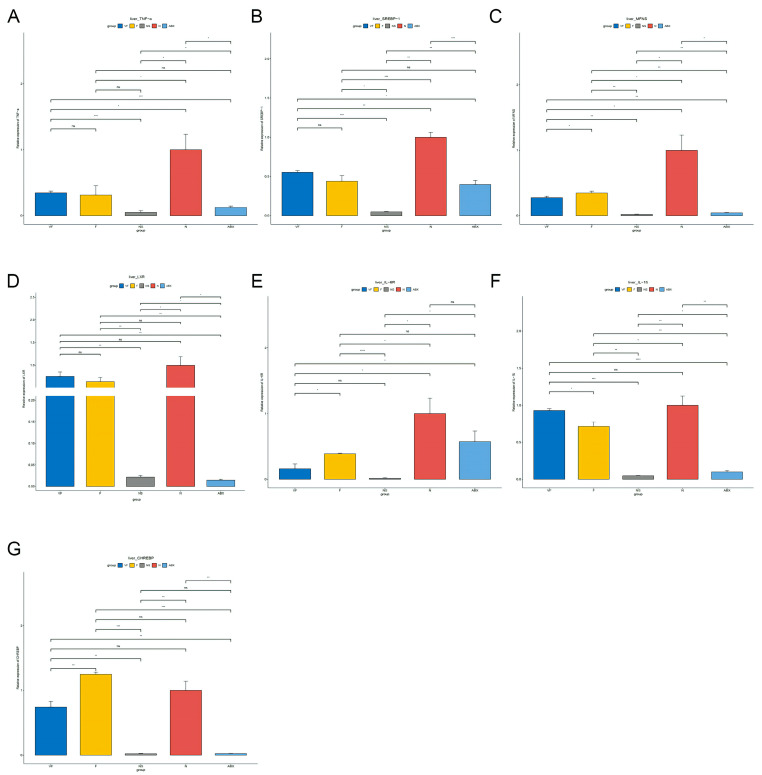
Gene qPCR results from different treated mice in the liver. (**A**) TNF-α, (**B**) Srebp-1, (**C**) Mfns, (**D**) LXR, (**E**) IL-6R, (**F**) IL-1β, and (**G**) Chrebp. (*: *p* < 0.05,**: *p* < 0.01,***: *p* < 0.001).

After vitamin C supplementation at the time of fecal transplantation, Mfns, IL-6R, and Chrebp decreased significantly and IL-1β increased significantly in the liver in the VF group compared to the F group. Srebp-1, LXR, and Chrebp were significantly decreased and TNF-α was significantly increased in intestinal tissues. Compared to the NS group, all genes except IL-6R were significantly increased in the liver and Chrebp was significantly decreased in the intestinal tissue of the VF group. TNF, Srebp-1, Mfns, and IL-6R were significantly decreased in the liver of the VF group compared to the N group. All five genes were significantly elevated in intestinal tissue except for TNF-α, IL-6R (Figure 11 and Figure 12).

**Figure 13:**
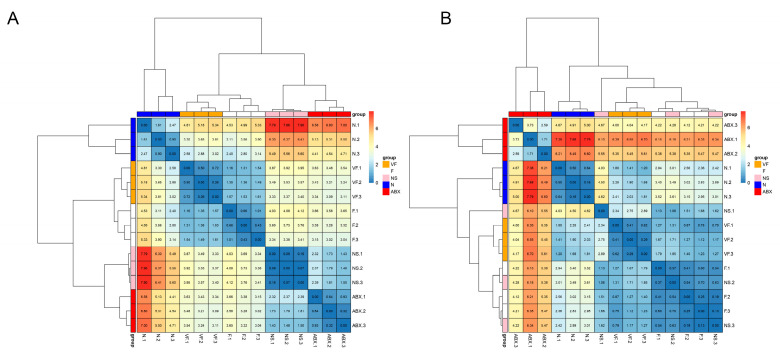
(**A**) Clustered heat map of gene expression in the small intestine. (**B**) Clustered heat map of gene expression in the liver.

The clustering results based on Euclidean distance showed that the VF group was the closest to the N group, both in the intestine and the liver (Figure 13).

## 4. Discussion

Antibiotic treatment can be detrimental to the gut microbiota as it kills some of the microorganisms, causing an imbalance in the microbial population. Prolonged use of antibiotics can further increase the risk of various diseases [31,32]. Moreover, antibiotics have long-term and subtle effects on organisms, including altering the gut microbiota. These effects are not limited to the gut alone. In fact, early exposure to antibiotics has been found to influence the lifespan of mice [29].

Vitamin C has been proven to be effective in treating a variety of diseases, including sepsis [33]. It acts as a cofactor for a range of biosynthetic and gene regulatory enzymes. Vitamin C can treat disease by improving gut microbiota [34] and is also used in high doses for cancer treatment [35]. On the other hand, vitamin C deficiency increases the risk of intestinal infections [36].

Studies have shown that vitamin C intake can significantly improve the homogeneity of the intestinal microbial community, reduce fecal pH, increase microbial α diversity, and fecal short-chain fatty acids [37]. Additionally, vitamin C acts as an antioxidant which directly impacts intestinal redox homeostasis, thus playing an important role in regulating the intestinal microbiota. This helps to correct the oxidative effects of antibiotics on gut microbes. Moreover, vitamin C protects the intestinal mucosa from damage and inflammation by enhancing the function of the intestinal epithelial barrier. A study conducted in Japan revealed that vitamin C intake was negatively associated with the risk of inflammatory bowel disease (IBD) [38].

Furthermore, vitamin C improves the intestinal micro-ecology by increasing the metabolites of probiotic genera such as alanine and lactate, while reducing the metabolites of intestinal pathogenic bacteria such as nitrates and nitrites. Our team supplemented fecal transplanted mice with vitamin C and observed that their gut microbiota profile was most similar to that of the donor mice. Additionally, the expression of genes in the intestine was closest to that of normal mice, further confirming the positive effect of vitamin C on the recovery of the gut microbiota.

The influence of gut microbiota on the immune system can be divided into two aspects: the innate immune system and the involvement of gut microbiota in the regulation of the body’s immune response. The colonization of gut microbiota early in life has an important influence on the development of the intestinal and systemic immune systems [39,40]. Dendritic cells play a critical role in regulating intestinal and systemic immunity [41]. Gut microbiota activates retinoic acid receptor α (RARα), forming the immunogenicity of human monocyte-derived dendritic cells, thereby regulating the immune response [42]. In addition, gut microbiota can also regulate immunity by affecting the intestinal barrier [43] and influencing gastric acid secretion.

Our study also looked at the effect of gut microbiota on immune-related genes after antibiotic treatment. The Cd4 molecule is known for its unique immune function [44] as it induces the selection of helper T cells during differentiation [45]. After disrupting the gut microbial ecology with antibiotics, we observed a significant up-regulation of CD4 expression in both the gut and liver, particularly in the gut where CD4 expression rose nearly thirty-fold. However, this returned to normal levels after receiving fecal microbial transplants, further demonstrating the impact of the gut microbiota on the immune system. The results obtained from our study demonstrate that Cd4, Ccr9, and Ccr3, which are associated with immunity and inflammation, along with Tenm4, a protein involved in neuronal connectivity, were significantly up-regulated in both the gut and liver after antibiotic (ABX) treatment. Conversely, Rgs16 and Lonrf3, which showed significant up-regulation in the intestine, displayed the opposite results in the liver. The relationship between gut microbiota and host gene expression is complex, with many interactions at play. The gut microbiota can directly influence the physiological metabolic processes of host cells through metabolites, short-chain fatty acids, and other factors, thus affecting host gene expression. Additionally, the gut microbiota can indirectly influence host gene expression by regulating the immune system and activating signaling pathways. These effects may lead to widespread changes in gene expression throughout the host’s body through various signaling cascades. Further research is necessary to gain more insight into the complex interactions between gut microbiota and host gene expression [46,47].

The time it takes for the gut microbiota to recover after oral antibiotics varies from person to person and depends on several factors such as individual differences, disease state, and diet. It can often take a considerable amount of time to fully recover. In our experiments, after 21 consecutive days of FMT treatment, mice showed improved but not complete return to normal levels of gut microbiota dysbiosis. Another dataset, PRJNA810918 [48], revealed that antibiotic-treated mice treated with FMT for eight consecutive weeks still had not returned to donor mouse levels of gut microbiota. Together, these two results suggest that the time needed for full recovery of the gut microbiota may exceed three months [49]. During the early stages of the COVID-19 epidemic, empirical antibiotic use resulted in patients’ gut microbiota not recovering even after the end of treatment [50]. However, the gut microbiota is variable, and one study showed that it responds rapidly to different diets. A specific diet in the short term can alter the community structure of the gut microbes, promoting diversity in dietary patterns [51].

The intestinal barrier plays a crucial role in regulating the passage of microorganisms, pro-inflammatory molecules, antigens, and toxins. Cytokines are a group of small molecule proteins with diverse biological activities that are synthesized and secreted by immune cells (such as monocytes, macrophages, T cells, B cells, NK cells, etc.) and certain non-immune cells (including endothelial cells, epidermal cells, fibroblasts, etc.) upon stimulation. Cytokines have powerful immunomodulatory effects and are essential for human physiology and pathology [52]. The research results showed that, among the various inflammatory markers tested (TNF-α, IL-1β, and IL-6R genes) in the liver, their expression was highest in the ABX group and lowest in the normal group. Moreover, IL-6 expression gradually decreased in the F and VF groups and the NS group. This suggests that IL-6 is significantly affected by antibiotics and the abundance of gut microbiota. Treatment with fecal transplants combined with vitamin C reduced the expression of the IL-6 cytokine gene in the community, which suggests that the inflammatory response is decreasing.

The side effects of taking antibiotics have been taken seriously, especially after a period of antibiotic abuse when many disadvantages became apparent. Continued use of antibiotics makes bacteria more resistant to them, rendering them ineffective against the bacteria. A significant proportion of microbial resistance results from mutations in genes related to the compound’s mechanism of action, or the acquisition of exogenous DNA through transformation, transduction, coupling, etc. [53]. Infections limited by multi-drug-resistant microorganisms that cannot be cured are becoming increasingly common in clinical practice. Antibiotic-associated diarrhea (AAD) is an example of the disruption of gut microbiota caused by antibiotics. Antibiotics not only kill harmful bacteria but also some beneficial ones, leading to dysbiosis of the gut microbiota and subsequent changes in carbohydrate, short-chain fatty acid, and bile acid metabolism, which can cause diarrhea. The prevalence of AAD is high in clinical settings, with estimates ranging from 5 to 39%, depending on the type and duration of antibiotic treatment [54]. The effects of dysbiosis on the ecology of the gut microbiota are widespread and long-lasting. In fact, more than half of antibiotic treatments are unnecessary [55], and to some extent, antibiotics become a new barrier to patient care. However, the inclusion of vitamin C as an adjunct to antibiotic therapy may be a promising new approach.

In contrast to previous studies, a distinctive feature of our approach is that we integrated public data to provide a more comprehensive analysis. Through this method we have investigated changes in the mice gut microbiome at the family and genus levels following oral administration of ampicillin and neomycin. Moreover, we demonstrated how these microbiome alterations affect the physiological function of the gut-liver axis through specific genetic changes. An additional innovation was the use of vitamin C as an adjuvant in efforts to facilitate the recovery of the altered gut microbiota, which yielded positive outcomes.

We recognize that this manuscript has some limitations. During the experiment, we refrained from establishing a specific daily dosage of antibiotics for the mice. This was attributed to our reliance on methodologies employed in prior research endeavors [30,56]. However, a precise administration of antibiotics would have enhanced the experiment’s interpretability and controllability. Regrettably, longitudinal sampling was absent, we conducted a comparison using group N mice as specimens. We utilized a small sample size for experimental validation and, for technical reasons, we used QIIME instead of QIIME 2 in our experiment. The genes we selected may not entirely reflect the impact of gut microbiota on gene expression in the small intestine and liver after oral antibiotic administration.

## 5. Conclusions

We investigated the impact of neomycin and ampicillin oral administration on gut microbiota composition in mice (f__Muribaculaceae decreasing, f__Lactobacillaceae increasing); genes related to gut-liver axis expression were significantly effected by this change in gut microbiota, but FMT with vitamin C supplementation was effective in restoring gut microbiota dysbiosis and gene expression.

## Data Availability

The raw Illumina read data were deposited in the NCBI Repository. Accession number: PRJNA879117.

## Supplementary Materials

The following supporting information can be downloaded at: https://www.mdpi.com/article/10.3390/genes14071423/s1, Supplementary Table S1: Dataset PRJDB6615 Relative abundance of bacterial flora at family level after oral antibiotics—5; Supplementary Table S2: Dataset PRJDB6615 Relative abundance of bacterial flora at genus level after oral antibiotics—6; Supplementary Table S3: Local data on relative abundance at family level after oral antibiotic dosing—5. Supplementary Table S4: Local data on relative abundance at family level after oral antibiotic dosing—6. Supplementary Figure S1: Comparison of gut microbiota α diversity between recipient FMT and donor mice in dataset PRJNA810918. Supplementary Table S5: qPCR primers. Supplementary Table S6: qPCR results in intestine. Supplementary Table S7: qPCR results in liver.

## References

[1] Gueimonde M., Sakata S., Kalliomäki M., Isolauri E., Benno Y., Salminen S. (2006). Effect of maternal consumption of lactobacillus GG on transfer and establishment of fecal bifidobacterial microbiota in neonates. J. Pediatr. Gastroenterol. Nutr..

[2] O’Hara A.M., Shanahan F. (2006). The gut flora as a forgotten organ. EMBO Rep..

[3] Song X., Wang L., Liu Y., Zhang X., Weng P., Liu L., Zhang R., Wu Z. (2022). The gut microbiota–brain axis: Role of the gut microbial metabolites of dietary food in obesity. Food Res. Int..

[4] Gomes A.C., Bueno A.A., de Souza R.G.M., Mota J.F. (2014). Gut microbiota, probiotics and diabetes. Nutr. J..

[5] Tao X., Wang N., Qin W. (2015). Gut Microbiota and Hepatocellular Carcinoma. Gastrointest. Tumors.

[6] Caesar R., Fåk F., Bäckhed F. (2010). Effects of gut microbiota on obesity and atherosclerosis via modulation of inflammation and lipid metabolism. J. Intern. Med..

[7] Buffie C.G., Jarchum I., Equinda M., Lipuma L., Gobourne A., Viale A., Ubeda C., Xavier J., Pamer E.G. (2012). Profound alterations of intestinal microbiota following a single dose of clindamycin results in sustained susceptibility to clostridium difficile-Induced colitis. Infect. Immun..

[8] Noverr M.C., Huffnagle G.B. (2005). The ‘microflora hypothesis’ of allergic diseases. Clin. Exp. Allergy.

[9] Engelbrektson A., Korzenik J.R., Pittler A., Sanders M.E., Klaenhammer T.R., Leyer G., Kitts C.L. (2009). Probiotics to minimize the disruption of faecal microbiota in healthy subjects undergoing antibiotic therapy. J. Med Microbiol..

[10] Wang J.-W., Kuo C.-H., Kuo F.-C., Wang Y.-K., Hsu W.-H., Yu F.-J., Hu H.-M., Hsu P.-I., Wang J.-Y., Wu D.-C. (2019). Fecal microbiota transplantation: Review and update. J. Formos Med. Assoc..

[11] Albesa I., Becerra M., Battán P.C., Páez P.L. (2004). Oxidative stress involved in the antibacterial action of different antibiotics. Biochem. Biophys. Res. Commun..

[12] Páez P.L., Becerra M.C., Albesa I. (2011). Comparison of macromolecular oxidation by reactive oxygen species in three bacterial genera exposed to different antibiotics. Cell Biochem. Biophys..

[13] Didier A.J., Stiene J., Fang L., Watkins D., Dworkin L.D., Creeden J.F. (2023). Antioxidant and Anti-Tumor Effects of Dietary Vitamins A, C, and E. Antioxidants.

[14] Romano K.A., Martinez-Del Campo A., Kasahara K., Chittim C.L., Vivas E.I., Amador-Noguez D., Balskus E.P., Rey F.E. (2017). Metabolic, Epigenetic, and Transgenerational Effects of Gut Bacterial Choline Consumption. Cell Host. Microbe..

[15] Wang X., Li Y., Chen W., Shi H., Eren A.M., Morozov A., He C., Luo G.-Z., Pan T. (2018). Transcriptome-wide reprogramming of N6-methyladenosine modification by the mouse microbiome. Cell Res..

[16] Kolodziejczyk A.A., Zheng D., Elinav E. (2019). Diet–microbiota interactions and personalized nutrition. Nat. Rev. Microbiol..

[17] Koch B.E.V., Yang S., Lamers G., Stougaard J., Spaink H.P. (2018). Intestinal microbiome adjusts the innate immune setpoint during colonization through negative regulation of MyD88. Nat. Commun..

[18] Ding J.-H., Jin Z., Yang X.-X., Lou J., Shan W.-X., Hu Y.-X., Du Q., Liao Q.-S., Xie R., Xu J.-Y. (2020). Role of gut microbiota via the gut-liver-brain axis in digestive diseases. World J. Gastroenterol..

[19] Arab J.P., Martin-Mateos R.M., Shah V.H. (2017). Gut–liver axis, cirrhosis and portal hypertension: The chicken and the egg. Hepatol. Int..

[20] Leclercq S., Matamoros S., Cani P.D., Neyrinck A.M., Jamar F., Stärkel P., Windey K., Tremaroli V., Bäckhed F., Verbeke K. (2014). Intestinal permeability, gut-bacterial dysbiosis, and behavioral markers of alcohol-dependence severity. Proc. Natl. Acad. Sci. USA.

[21] Lang S., Duan Y., Liu J., Torralba M.G., Kuelbs C., Ventura-Cots M., Abraldes J.G., Bosques-Padilla F., Verna E.C., Brown R.S. (2020). Intestinal fungal dysbiosis and systemic immune response to fungi in patients with alcoholic hepatitis. Hepatology.

[22] Mouzaki M., Comelli E.M., Arendt B.M., Bonengel J., Fung S.K., Fischer S.E., McGilvray I.D., Allard J.P. (2013). Intestinal microbiota in patients with nonalcoholic fatty liver disease. Hepatology.

[23] Bajaj J.S., Heuman D.M., Hylemon P.B., Sanyal A.J., White M.B., Monteith P., Noble N.A., Unser A.B., Daita K., Fisher A.R. (2014). Altered profile of human gut microbiome is associated with cirrhosis and its complications. J. Hepatol..

[24] Dapito D.H., Mencin A., Gwak G.-Y., Pradere J.-P., Jang M.-K., Mederacke I., Caviglia J.M., Khiabanian H., Adeyemi A., Bataller R. (2012). Promotion of hepatocellular carcinoma by the intestinal microbiota and TLR4. Cancer Cell.

[25] Schnabl B., Brenner D.A. (2014). Interactions between the intestinal microbiome and liver diseases. Gastroenterology.

[26] Segata N., Izard J., Waldron L., Gevers D., Miropolsky L., Garrett W.S., Huttenhower C. (2011). Metagenomic biomarker discovery and explanation. Genome Biol..

[27] Caporaso J.G., Kuczynski J., Stombaugh J., Bittinger K., Bushman F.D., Costello E.K., Fierer N., Gonzalez Peña A., Goodrich J.K., Gordon J.I. (2010). QIIME allows analysis of high-throughput community sequencing data. Nat. Methods.

[28] Ward T., Larson J., Meulemans J., Hillmann B., Lynch J., Sidiropoulos D., Spear J.R., Caporaso G., Blekhman R., Knight R. (2017). BugBase predicts organism-level microbiome phenotypes. BioRxiv.

[29] Lynn M.A., Eden G., Ryan F.J., Bensalem J., Wang X., Blake S.J., Choo J.M., Chern Y.T., Sribnaia A., James J. (2021). The composition of the gut microbiota following early-life antibiotic exposure affects host health and longevity in later life. Cell Rep..

[30] Blake S.J., James J., Ryan F.J., Caparros-Martin J., Eden G.L., Tee Y.C., Salamon J.R., Benson S.C., Tumes D.J., Sribnaia A. (2021). The immunotoxicity, but not anti-tumor efficacy, of anti-CD40 and anti-CD137 immunotherapies is dependent on the gut microbiota. Cell Rep. Med..

[31] Scott F.I., Horton D.B., Mamtani R., Haynes K., Goldberg D.S., Lee D.Y., Lewis J.D. (2016). Administration of Antibiotics to Children Before Age 2 Years Increases Risk for Childhood Obesity. Gastroenterology.

[32] Tsakok T., McKeever T., Yeo L., Flohr C. (2013). Does early life exposure to antibiotics increase the risk of eczema? A systematic review. Br. J. Dermatol..

[33] Marik P.E. (2018). Vitamin C for the treatment of sepsis: The scientific rationale. Pharmacol. Ther..

[34] Li Y., Zafar S., Ibrahim R.M.S., Chi H.-L., Xiao T., Xia W.-J., Li H.-B., Kang Y.-M. (2021). Exercise and food supplement of vitamin C ameliorate hypertension through improvement of gut microflora in the spontaneously hypertensive rats. Life Sci..

[35] Mussa A., Idris R.A.M., Ahmed N., Ahmad S., Murtadha A.H., Din T.A.D.A.A.T., Yean C.Y., Rahman W.F.W.A., Lazim N.M., Uskoković V. (2022). High-Dose Vitamin C for Cancer Therapy. Pharmaceuticals.

[36] Moya-Alvarez V., Koyembi J.J., Kayé L.M., Mbecko J., Sanke-Waîgana H., Djorie S.G., Nyasenu Y.T., Mad-Bondo D., Kongoma J., Nakib S. (2021). Vitamin C levels in a Central-African mother–infant cohort: Does hypovitaminosis C increase the risk of enteric infections?. Matern. Child Nutr..

[37] Pham V.T., Fehlbaum S., Seifert N., Richard N., Bruins M.J., Sybesma W., Rehman A., Steinert R.E. (2021). Effects of colon-targeted vitamins on the composition and metabolic activity of the human gut microbiome—A pilot study. Gut Microbes.

[38] Sakamoto N., Kono S., Wakai K., Fukuda Y., Satomi M., Shimoyama T., Inaba Y., Miyake Y., Sasaki S., Okamoto K. (2005). Dietary risk factors for inflammatory bowel disease: A multicenter case-control study in Japan. Inflamm. Bowel Dis..

[39] Kelly D., King T., Aminov R. (2007). Importance of microbial colonization of the gut in early life to the development of immunity. Mutat. Res. Mol. Mech. Mutagen..

[40] Gensollen T., Iyer S.S., Kasper D.L., Blumberg R.S. (2016). How colonization by microbiota in early life shapes the immune system. Science.

[41] Luciani C., Hager F.T., Cerovic V., Lelouard H. (2022). Dendritic cell functions in the inductive and effector sites of intestinal immunity. Mucosal Immunol..

[42] Bene K., Varga Z., Petrov V.O., Boyko N., Rajnavolgyi E. (2017). Gut Microbiota Species Can Provoke both Inflammatory and Tolerogenic Immune Responses in Human Dendritic Cells Mediated by Retinoic Acid Receptor Alpha Ligation. Front. Immunol..

[43] Wang Y., Shi Y., Li W., Wang S., Zheng J., Xu G., Li G., Shen X., Yang J. (2022). Gut microbiota imbalance mediates intestinal barrier damage in high-altitude exposed mice. FEBS J..

[44] Mahnke Y.D., Brodie T.M., Sallusto F., Roederer M., Lugli E. (2013). The who’s who of T-cell differentiation: Human memory T-cell subsets. Eur. J. Immunol..

[45] Singer A., Bosselut R. (2004). CD4⧸CD8 Coreceptors in thymocyte development, selection, and lineage commitment: Analysis of the CD4⧸CD8 lineage decision. Adv. Immunol..

[46] Greenhill C. (2016). Intestinal microbiota affects host physiology. Nat. Rev. Endocrinol..

[47] Aktar R., Parkar N., Stentz R., Baumard L., Parker A., Goldson A., Brion A., Carding S., Blackshaw A., Peiris M. (2020). Human resident gut microbe *Bacteroides thetaiotaomicron* regulates colonic neuronal innervation and neurogenic function. Gut Microbes.

[48] Xu L., Zhang Q., Dou X., Wang Y., Wang J., Zhou Y., Liu X., Li J. (2022). Fecal microbiota transplantation from young donor mice improves ovarian function in aged mice. J. Genet. Genom..

[49] Tian Y., Sun K.-Y., Meng T.-Q., Ye Z., Guo S.-M., Li Z.-M., Xiong C.-L., Yin Y., Li H.-G., Zhou L.-Q. (2021). Gut Microbiota May Not Be Fully Restored in Recovered COVID-19 Patients After 3-Month Recovery. Front. Nutr..

[50] Zuo T., Zhang F., Lui G.C.Y., Yeoh Y.K., Li A.Y.L., Zhan H., Wan Y., Chung A.C.K., Cheung C.P., Chen N. (2020). Alterations in Gut Microbiota of Patients With COVID-19 During Time of Hospitalization. Gastroenterology.

[51] David L.A., Maurice C.F., Carmody R.N., Gootenberg D.B., Button J.E., Wolfe B.E., Ling A.V., Devlin A.S., Varma Y., Fischbach M.A. (2014). Diet rapidly and reproducibly alters the human gut microbiome. Nature.

[52] Zheng X., Wu Y., Bi J., Huang Y., Cheng Y., Li Y., Wu Y., Cao G., Tian Z. (2022). The use of supercytokines, immunocytokines, engager cytokines, and other synthetic cytokines in immunotherapy. Cell. Mol. Immunol..

[53] Munita J.M., Arias C.A. (2016). Mechanisms of Antibiotic Resistance. Microbiol. Spectr..

[54] McFarland L. (1998). Epidemiology, Risk Factors and Treatments for Antibiotic-Associated Diarrhea. Dig. Dis..

[55] Bassetti S., Tschudin-Sutter S., Egli A., Osthoff M. (2022). Optimizing antibiotic therapies to reduce the risk of bacterial resistance. Eur. J. Intern. Med..

[56] Miyauchi E., Kim S.-W., Suda W., Kawasumi M., Onawa S., Taguchi-Atarashi N., Morita H., Taylor T.D., Hattori M., Ohno H. (2020). Gut microorganisms act together to exacerbate inflammation in spinal cords. Nature.

