# Supplementary figures and images for: Antibiotic-Induced Dysbiosis of the Gut Microbiota Impairs Gene Expression in Gut-Liver Axis of Mice
Source: Genes (Basel). 2023 Jul 10;14(7):1423. doi: 10.3390/genes14071423 (PMC10379678; doi:10.3390/genes14071423)

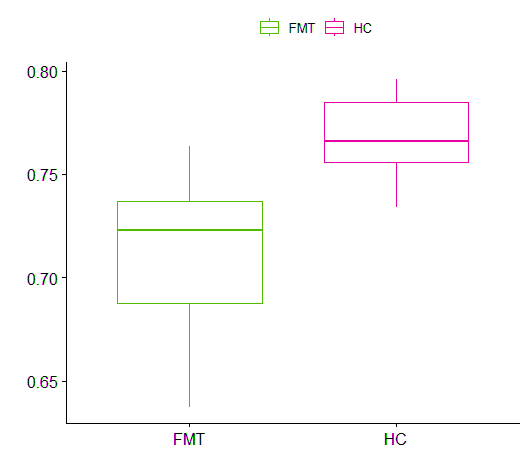

Supplement: Supplementary file 1 [file genes-14-01423-s001.zip › Figure S1.Comparison of gut microbiota alpha diversity between recipient FMT and donor mice in dataset PRJNA810918.png]
